# Minor lipid components of some *Acacia* species: potential dietary health benefits of the unexploited seeds

**DOI:** 10.1186/1476-511X-11-49

**Published:** 2012-05-11

**Authors:** Nizar Nasri, Walid Elfalleh, Nizar Tlili, Hédia Hannachi, Saida Triki, Abdelhamid Khaldi

**Affiliations:** 1 Laboratoire de Biochimie, Campus Universitaire, Tunis, 2092, Tunisia; 2 Institut des Régions Arides, Laboratoire d’Aridoculture et Cultures Oasiennes, Médenine, 4119, Tunisia; 3 Banque Nationale de Gènes, Charguia-1, Tunis, 1080, Tunisia; 4 Institut National de Recherches en Génie Rural Eaux et Forêts, P.B. 10, Ariana, 2080, Tunisia

**Keywords:** Unexploited *Acacia*, Oilseeds, Carotenoids, Tocopherols, Sterols

## Abstract

**Background:**

Oilseed samples from four *Acacia* species ( *A. cyclops*, *A. ligulata*, *A. salicina* and *A. cyanophylla*) were analyzed in order to evaluate the potential nutritional value of their unexploited seeds.

**Methods:**

Samples were collected from different Tunisian geographic locations. Seed oils were extracted and carotenoids, tocopherols and sterols were analyzed using chromatographic methods.

**Results:**

The studied *Acacia* seeds seem to be quite rich in lipids (from 6% to 12%). All *Acacia* species contain mainly the xanthophylls zeaxanthin and lutein compounds: from ca. 38 mg.kg^-1^ of total lipids (*A. cyclops)* to ca. 113 mg.kg^-1^ of total lipids *(A. cyanophylla)*. Total tocopherols varied from ca. 221 mg.kg^-1^ of total lipids (*A. cyclops*) to ca. 808 mg.kg^-1^ of total lipids (*A. ligulata*). Sterols are highly present and their contents ranged between ca. 7 g. kg^-1^ of total lipids (*A. salicina*) and 11 g. kg^-1^ of total lipids (*A. cyclops*).

**Conclusion:**

This study highlights that these unexploited seeds might have a potential nutritional value and encourages researchers to more explore and find developments for these plants for healthy purposes.

## Introduction

Currently, worldwide interest is oriented for the recovery and exploitation of oils from natural plant resources. Vegetable oils with a high relative amount of minor lipid components are of great importance for human health [1].

Plant sterols (phytosterols) are natural dietary components with serum cholesterol-lowering proprieties. Sterols are a group of fundamental compounds of cell membranes in both plants and animals. The most common plant sterols are β-sitosterol, campesterol, and stigmasterol, which are classified as *4-*desmethylsterols of the cholestane series [2]. The structures of plant sterols are similar to that of cholesterol with an extra methyl or ethyl group and a double bond in the side chain. Unlike cholesterol, they are not synthesized by the human body and are minimally absorbed from the gut [3]. The exact mechanism of their cholesterol lowering properties is not fully understood, but plant sterols appear to inhibit the uptake of dietary and biliary cholesterol from the distal small intestine by competing with cholesterol for incorporation into mixed micelles [4]. Plant sterol and stanol-enriched spreads are now widely available commercially as functional foods, but also have specific potential uses in clinical practice. Plant sterols are important ingredients of the blended functional oil [5]. Tocopherols are considered to be the most effective lipid phase natural antioxidants. They prevent lipid peroxidation by acting as peroxyl radical scavengers that terminate chain reactions in membranes and lipoprotein particles. The role of tocopherols in cellular signaling, especially in relation to protein kinase C was also confirmed [6]. Carotenoids are fat soluble compounds that are associated with the lipidic fractions [7]. Carotenoids are synthesized by plants and many microorganisms. They are recognized mainly as natural antioxidants and enhancers of the immune response [8]. Recently, these properties have increased the interest on the analysis of carotenoids in vegetable samples.

Nowadays, plant seeds constitute new oil sources, especially from underexploited seeds such as *Acacia* genus. Little is known about the chemistry of most *Acacia* species, although the genus is quite large and widespread in the warm sub-arid and arid portions of the world [9]. The *Acacia* genus comprises approximately 1350 species [10]. The present paper is to investigate, for the first time the carotenoids, tocopherols and sterols from seeds of some Tunisian *Acacia* species ( *A. cyclopsA. ligulataA. salicina* and *A. cyanophylla*). The potential dietary importance of their unexploited seeds is discussed.

## Materials and methods

### Materials

Samples from fully mature fruits were collected in June 2010 and used in the present study. *Acacia* seeds were harvested from four species found in Tunisia, respectively *A. cyclops*, *A. ligulata*, *A. salicina* and *A. cyanophylla*.

### Chemicals

All solvents used during the experiments (methanol, dichloromethane) were purchased from Fluka (Ridel-de Haën, Switzerland). 5α-cholestane, lutein, zeaxanthin, α-tocopherol, γ-tocopherol and δ-tocopherols were purchased from Sigma-Aldrich (Steinheim, Germany). Petroleum ether, potassium hydroxide pellets and anhydrous sodium sulphate were obtained from Merck (Darmstadt, Germany).

### Oil extraction

The oil content was determined according to the reference [11]. About 20 g of *Acacia* seeds was ground in a mortar and extracted using petroleum ether in a Soxhlet apparatus for 6 h. The solvent was concentrated using a rotary evaporator under reduced pressure at 45°C. The oil was dried under a nitrogen stream and stored at −20°C until use. To minimize the decomposition and oxidation of the extracted compounds, all samples were collected in brown glass bottles to prevent UV-activated degradation. All analyses were performed in triplicate.

### Extraction of lutein, zeaxanthin and tocopherols

40 mg of oil was resuspended in 1 mL of a mixed HPLC mobile phase: acetonitrile/methanol at 50 mM, and ammonium acetate/water/dichloromethane (700:150:50:100, by vol.). After resuspension, the extract was vortexed for 30 s. Samples of 80 μL were injected into the HPLC system for the analysis of lutein and zeaxanthin.

### HPLC analysis of lutein, zeaxanthin and tocopherols

The HPLC apparatus was a Jasco PU-1580 Plus intelligent pump equipped with an automatic injector system AS300 (Thermo Finnigan, les Ulis, France) and a Jasco MD-1510 plus multi-wavelength detector (JASCO International Co., Ltd., Japan). HPLC analyses were carried out using RP-HPLC with a Nucleosil C18 column (25 mm x 4.6 mm id, 5 μm particle size) and a VIDAK C18 column (25 mm x 4.6 mm id, 5 μm particle size). The analytical conditions were based on those reported by [Lyan et al. 12], with some modifications: Isocratic solvent system; acetonitrile/methanol at 50 mM ammonium acetate/water/dichloromethane (700:150:50:100, by vol.); flow rate = 2 mL.min^-1^, and detection at 450 nm for lutein and zeaxanthin and 298 nm for tocopherols.

### Identification of lutein and zeaxanthin

The identification of lutein, zeaxanthin and tocopherols was ensured by comparing the retention times and absorption spectra of unknown peaks with those of reference standards and by adding lutein, zeaxanthin, α-, β-, δ-tocopherol standards to the sample for co-chromatography.

### Preparation of the standard curve

Six quantities of lutein (range: 0.125–5 μg), zeaxanthin (range: 50–300 ng), α-tocopherol (range: 0.5–40 μg), γ-tocopherol (range: 1–25 μg) and δ-tocopherol (range: 0.1–2 μg) were injected into the HPLC system (each standard being dissolved in 1 mL of the HPLC mobile phase: acetonitrile/methanol at 50 mM ammonium acetate/water/dichloromethane (700:150:50:100, by vol.). The linear regression equation for each standard curve was then obtained by plotting the amount of the standard compound injected against the peak surface area. The regression equation and correlation coefficient (*r*^2^) were calculated using ChromNav software (JASCO).

### Sterol extraction

A mixture of 50 mg of seed oil, 25 μL 5α cholestane (1 mg.mL^-1^) used as an internal standard and 5 mL methanolic KOH (1 N) was saponified in a capped flask for 16 h at room temperature. 10 mL distilled water and 10 mL dichloromethane were then added and mixed. The resulting solution was centrifuged and the lower fraction was kept in a second capped flask. The upper organic layers were washed twice with 10 mL distilled water, and once with 10 mL dichloromethane. The resulting solution was centrifuged and the dichloromethane layers were combined and washed twice with 5 mL and kept in the second capped flask. This solution was filtered and the obtained solvent was evaporated to dryness under nitrogen at 40°C. After vortexing, the aliquot (matter unsaponifiable with dichloromethane) was derivatized to trimethylsilyl ethers (TMS ether) by the addition of 300 μL *N,O-bis*(trimethylsilyl)trifluoroacetamide and 50 μL pyridine at 60°C for 30 min, and then injected into the gas chromatograph.

### Sterol quantification by gas chromatography-flame ionization detection (GC–FID)

Samples (2 μL) were analyzed in duplicate by GC in a Hewlett-Packard HP-4890D chromatograph equipped with a 30 m (0.25 mm i.d., 0.25-μm film thickness) DB5 MS fused silica capillary column. The oven temperature was raised from 50°C to 290°C at a rate of 20°C min^-1^. The flame ionization detector (FID) temperature was 290°C. The split ratio was 1:20. Helium was used as a carrier gas at a pressure of 120 kPa. TMS esters were eluted from the column. The data were processed using EZChrom Elite software (Agilent Technologies, Massy, France). The areas of both sterols were compared to the areas of known quantities of the internal standard (5α-cholestan).

### Sterol identification by gas chromatography–mass spectrometry (GC-MS)

GC-MS analyses of TMS ester derivatives were carried out on a Shimadzu GC 2010 gas chromatograph attached to a Shimatdzu 2010 selective quadrupole mass detector (Shimadzu France, Marne la Vallée) operating in the electronic ionisation mode under an ionisation voltage of 70 eV at 200°C. Shimadzu software was used for data acquisition and processing. The injector (splitless mode) and the interface temperature were maintained at 290°C; helium was used as the carrier gas under a constant flow rate of 1 mL/min. Spectral data were acquired over a mass range of 50–600 amu. GC separation was performed on a DB5 MS fused silica capillary column (0.25 mm i.d., 0.25-μm film thickness). The temperature was kept at 50°C for 1 min, and then raised to 290°C for 90 min at a rate of 20°C min^-1^.

### Statistical and chemometric methods

Data were compared on the basis of standard deviations from mean values. Differences between mean values were based on the one-way analysis of variance with a post-hoc determination using Duncan’s multiple range tests performed by Statistica software (version 8). The level of significance was set at *p* <0.05.

## Results and discussion

### Oil content

Total lipid contents, expressed as percentage on dry weight basis (dw%) have a values of 6.83 dw % (*A. cyclops*), 9.03 dw% ( *A. ligulata*), 10.05 dw% ( *A. cyanophylla*) and 12.18 dw% ( *A. salicina*) (Table 1). The studied *Acacia* species seem to be quite rich in lipids (from 6 to 12%) and are well comparable to other species [13-17].

**Table 1:** **Carotenoids and tocopherols**^**†**^**(mg.kg**^**-1**^**of total lipids) of*Acacia*seed oils**

**Species**	**Oil content (dw%)**	**Carotenoids**			**Tocopherols**			**Total tocopherols**
		**Lutein**	**Zeaxanthin**	**Total carotenoids**	**α tocopherol**	**γ tocopherol**	**δ tocopherol**	
*Acacia ligulata*	9.03	43.83 ^BC^	6.28 ^A^	50.10	315.85 ^B^	462.14 ^A^	30.77 ^A^	808.76
*Acacia cyclops*	6.83	35.41 ^C^	2.80 ^B^	38.21	85.51 ^C^	127.25 ^D^	8.66 ^B^	221.42
*Acacia salicina*	12.18	54.67 ^B^	2.37 ^B^	57.04	404.30 ^B^	155.78 ^C^	3.33 ^C^	563.41
*Acacia cyanophylla*	10.05	109.55 ^A^	4.21 ^A^	113.76	560.14 ^A^	185.41 ^B^	9.15 ^B^	754.70

^†^ Each value is the mean of duplicate analyses.

Superscript letters with different letters in the same column of species indicate a significant difference (*p* <0.05) analyzed using Duncan’s multiple range test.

### Carotenoid and tocopherol contents

All *Acacia* species contain mainly the xanthophylls zeaxanthin and lutein compounds as reported in Table 1. Total amounts of xanthophylls were respectively for *A. cyclops*, *A. ligulata*, *A. salicina* and *A. cyanophylla*: 38.21 mg.kg^-1^ TL (Total Lipids), 50.10 mg.kg^-1^ TL, 57.04 mg.kg^-1^ TL, and 113.76 mg.kg^-1^ TL. Contents of the main compound (lutein) were 35.41 mg.kg^-1^ TL (*A. cyclops*), 43.83 mg.kg^-1^ TL (*A. ligulata*), 54.67 mg.kg^-1^ TL (*A. salicina*) and 109.55 mg.kg^-1^ TL (*A. cyanophylla*). These differences are mainly due to genetic factors. Highly amounts of carotenoids make the genus Acacia a good natural source of these compounds, especially luteins.

As for tocopherols, all *Acacia* species contain mainly α- and γ-tocopherols and in some few amounts the δ-tocopherol. Table 1. The absence of ß-tocopherol was confirmed using the same method previously described. Regarding α and γ tocopherol contents for the different studied *Acacia*, we noticed that α-tocopherol is the major compound for *A. salicina* (404.30 mg.kg^-1^ TL) and *A. cyanophylla* (560.14 mg.kg^-1^ TL). As for as *A. cyclops* and *A. ligulata*, γ-tocopherols was found to be the major compound, with respectively 127.25 mg.kg^-1^ TL and 462.14 mg.kg^-1^ TL. For all *Acacia*, the total tocopherols ranged between 221.42 mg.kg^-1^TL (*Acacia cyclops*) and 808.76 mg.kg^-1^ TL (*Acacia ligulata*). Tocopherols from all studied *Acacia* were highly presented compared to some other vegetable oils like grape seed (142.6 mg.kg^-1^ TL) and are comparable to those of olive (216.8 mg.kg^-1^), flaxseed (588.5 mg.kg^-1^), peanut (398.6 mg.kg^-1^), pumpkin (508.1 mg.kg^-1^ TL), rapeseed (624.6 mg.kg^-1^ TL), and sunflower (634.4 mg.kg^-1^ TL). But these contents are lower than soybean (1797.6 mg.kg^-1^ TL) or maize (1618.4 mg.kg^-1^ TL), for example [18].

### Sterol content

Sterol contents of the studied *Acacia* are respectively 7.33 g. kg^-1^ TL (*A. salicina*), 7.70 g.kg^-1^ TL (*A. ligulata*) 8.94 g. kg^-1^ TL (*A. cyanophylla*) and 11.62 g.kg^-1^ TL (*A. cyclops*). All of these contents are higher than soybean (1.61 g kg^-1^), almond (1.43 g kg^-1^ TL), olive oil (2.21 g kg^-1^ TL), or peanut (2.2 g kg^-1^ TL), but still comparable to those of sesame oil (8.65 mg kg^-1^ TL) or corn oil (9.68 mg kg^-1^ TL). New findings are further confirmation of the high nutritional value of the genus *Acacia,* since sterols are known to decrease the risk of certain types of cancer and enhance immune function [19]. The sterols are also known to reduce serum low-density lipoprotein (LDL)-cholesterol level, and food products containing these plant compounds are widely used as a therapeutic dietary option to reduce plasma cholesterol and atherosclerotic risk [20]. For all *Acacia* species, β sitosterol was the major compound (between 45.5% and 53.9%), followed by the Δ7 stigmastenol (between 14.4% and 22.1%). All other sterols are present with amounts lower than 5.8% (Table 2). To our knowledge, very few studies were established to evaluate sterols from *Acacia* species. The phytosterols α-spinasterol and stigmast-7-enol have been characterized from *A. auriculiformis*[21]. Many of these species also contained 5α-stigmastanol, β-sitosterol, and stigmasterol [9,22].

**Table 2:** **Sterols (g. kg**^**-1**^**of total lipids) of*Acacia*seed oils**

**Phytosterols**	**Species**			
	***Acacia ligulata***	***Acacia cyclops***	***Acacia salicina***	***Acacia cyanophylla***
Cholesterol	*tr*^††^	0.22 ^**A**^	0.06 ^**C**^	0.07 ^**B**^
^Δ7^ cholestenol	*tr*	0.03 ^**A**^	0.06 ^**A**^	*tr*
Campesterol	0.08 ^**A**^	0.20 ^**A**^	0.13 ^**A**^	0.20 ^**A**^
Campestanol	0.20 ^**C**^	0.42 ^**A**^	0.11 ^**D**^	0.27 ^**B**^
Stigmasterol	0.31 ^**B**^	0.36 ^**A**^	0.15 ^**C**^	0.29 ^**B**^
^Δ7^ stigmasterol	0.28 ^**C**^	0.27 ^**C**^	0.57 ^**A**^	0.36 ^**B**^
^Δ7^ campesterol	0.35 ^**C**^	*tr*	0.38 ^**B**^	0.45 ^**A**^
^Δ 5,23^ stigmastadienol	0.19 ^**A**^	0.05 ^**B**^	0.15 ^**A,B**^	0.22 ^**A**^
β sitosterol	4.15 ^**B**^	5.40 ^**A**^	3.48 ^**C**^	4.06 ^**B**^
^Δ5^ avenasterol	0.24 ^**C**^	1.13 ^**A**^	0.38 ^**B**^	0.33 ^**B,C**^
^Δ7^ sitosterol	*tr*	*tr*	0.21 ^**A**^	0.20 ^**A**^
^Δ 5, 24 (25)^ stigmasterol	0.09 ^**C**^	0.18 ^**A**^	0.12 ^**B**^	0.20 ^**A**^
^Δ5, 24^ stigmastadienol	0.21 ^**B**^	0.23 ^**A**^	0.08 ^**C**^	*tr*
^Δ7^ stigmastenol	1.11 ^**C**^	2.57 ^**A**^	1.22 ^**C**^	1.78 ^**B**^
Cycloartenol	0.45 ^**A**^	0.38 ^**B**^	0.15 ^**D**^	0.33 ^**C**^
^Δ7^ avenasterol	0.02 ^**B**^	0.16 ^**A**^	0.03 ^**B**^	0.05 ^**B**^
^24, methylene^ cycloartanol	0.02 ^**A,B**^	*tr*	0.05 ^**A**^	0.06 ^**A**^
Citrostadienol	0.02 ^**A**^	*tr*	tr	0.05 ^**A**^
**Total phytosterols**	**7.70**	**11.62**	**7.33**	**8.94**

^†^ Each value is the mean of duplicate analyses.

^††^*tr*: traces.

Superscript letters with different letters in the same line of species indicate a significant difference (*p* <0.05) analyzed using Duncan’s multiple range test.

## Conclusion

The studied *Acacia*s seem to be quite rich in lipids (from 6 to 12%) and are well comparable to other *Acacia* species. The composition of *Acacia* species lipid fraction is reported here for the first time. Studied *Acacia* species contain very high levels of carotenoids, tocopherols and sterols. Carotenoids from studied *Acacias* reached 113 mg.kg^-1^ TL and tocopherols reached 808 mg.kg^-1^ TL. Sterols reached 11 g.kg^-1^ TL. As these minor compounds are known to have a wide range of beneficial biological activities and physical properties, the oil from *Acacia* seeds confirms its nutritional value and dietary importance. This study explores that these unexploited seeds might replace conventional oil types such sunflower or rapeseed oils.

## Competing interests

The authors declare that they have no competing interests.

## Authors’ contributions

NN performed experimental work, interpretation and discussion of the results and wrote the paper. WE, NT, HH, ST, KA conceived drafting and revision of the manuscript. All authors read and approved the final manuscript.

## References

[B1] Carvalho IS Miranda I Pereira H Evaluation of oil composition of some crops suitable for human nutrition Ind Crop Prod 2006 24 75 78 10.1016/j.indcrop.2006.03.005

[B2] Kochar SP Influence of processing on sterols of edible vegetable oils Prog Lipid Res 1983 22 161 188 10.1016/0163-7827(83)90008-5 6356149

[B3] Pollak OJ Kritchevsky D Monographs in Atherosclerosis 1981 Basel, New York 7231433

[B4] Ling WH Jones PJH Minireview dietary phytosterols: a review of metabolism, benefits and side effects Life Sci 1995 57 195 206 10.1016/0024-3205(95)00263-6 7596226

[B5] St-Onge MP Lamarche B Mauger JF Jones PJH Consumption of a functional oil rich in phytosterols and medium-chain triglyceride oil improves plasma lipid profiles in men J Nutr 2003 133 1815 1820 12771322 10.1093/jn/133.6.1815

[B6] Azzi A Aratri E Boscoboinik D Clement S Ozer NK Ricciarelli R Spycher S Molecular basis of alpha-tocopherol control of smooth muscle cell proliferation Biofactors 1998 7 3 14 10.1002/biof.5520070102 9523023

[B7] de Quiros AR-B Costa HS Analysis of carotenoids in vegetable and plasma samples: A review J Food Compos Anal 2006 19 97 111 10.1016/j.jfca.2005.04.004

[B8] van den Berg H Faulks R Granado HF Hirschberg J Olmedilla B Sandmann G Southon S Stahl W The potential for the improvement of carotenoid levels in foods and the likely systemic effects J Sci Food Agr 2000 80 880 912 10.1002/(SICI)1097-0010(20000515)80:7<880::AID-JSFA646>3.0.CO;2-1

[B9] Seigler DS Phytochemistry of Acacia—sensu lato Biochem Syst Ecol 2003 31 845 873 10.1016/S0305-1978(03)00082-6

[B10] Maslin BR Miller JT Seigler DS Overview of the generic status of Acacia (Leguminosae: Mimosoideae) Aust Syst Bot 2003 16 1 18 10.1071/SB02008

[B11] ISO, International Organization for Standardization ISO 659:1998 Oil seeds determination of hexane extract (or light petroleum extract), called “oil content” 1999 ISO, Geneva

[B12] Lyan B Azais-Braesco V Cardinault N Tyssandier V Borel P Alexandre-Gouabau MC Grolier P A simple method for clinical determination of 13 carotenoids in human plasma using an isocratic HPLC method J Chromatogr B 2001 751 297 303 10.1016/S0378-4347(00)00488-6 11236085

[B13] Akthar S Chemical and nutritional evaluation of Genus Acacia of the family Leguminosae of pakistan 1992 Thesis, Institute of Chemistry/University of the Punjab, Pakistan 240pp

[B14] Youzbachi N Elfalleh W Tlili N Gregoire S Berdeaux O Salles C Triki S Khouja ML Khaldi A Nasri N Unexploited Acacia cyanophylla seeds: potential food sources of ω6 fatty acids and antioxidants? J Sci Food Agr 2012 92 1526 1532 10.1002/jsfa.4737 22228365

[B15] Paganuzzi V Malerba A Composition of Acacia salicina Lindl seed oil Comparison with seed oils of other Acacias species Riv Ital Sostanze Gr 2000 77 597 607

[B16] Glyphis JP Milton SJ Siegfried WR Dispersal of Acacia Cyclops by birds Oecologia 1981 48 138 141 10.1007/BF00347002 28309947

[B17] Lamarque AL Fortunato RH Maestri DM Guzman CA Seed components and taxonomy of some species Biochem Syst Ecol 2000 28 53 60 10.1016/S0305-1978(99)00037-X

[B18] Tuberoso CIG Kowalczyk A Sarritzu E Cabras P Determination of antioxidant compounds and antioxidant activity in commercial oilseeds for food use Food Chem 2007 103 1494 1501 10.1016/j.foodchem.2006.08.014

[B19] Bouic PJ The role of phytosterols and phytosterolins in immune modulation: a review of the past 10 years Curr Opin Clin Nutr Metab Care 2001 4 471 475 10.1097/00075197-200111000-00001 11706278

[B20] Calpe-Berdiel L Escol-Gil JC Blanco-Vaca F New insights into the molecular actions of plant sterols and stanols in cholesterol metabolism Atherosclerosis 2009 203 18 31 10.1016/j.atherosclerosis.2008.06.026 18692849

[B21] Mahato SB Pal BC Price KR Structure of acaciaside, a triterpenoid trisaccharide from Acacia auriculiformis Phytochem 1989 28 207 210 10.1016/0031-9422(89)85039-3

[B22] Clark-Lewis JW Dainis I The phytosterols from Acacia species: α-spinasterol and stigmast-7-enol Aust J Chem 1967 20 1961 1974 10.1071/CH9671961

